# Digital neurosurgery in the era of intelligent medicine: a scoping review

**DOI:** 10.3389/fmed.2025.1700166

**Published:** 2025-12-16

**Authors:** Zhengbo Yuan, Zhongjie Shi, Zhanxiang Wang

**Affiliations:** 1 Department of Neurosurgery, The First Affiliated Hospital of Xiamen University, School of Medicine, Xiamen University, Xiamen, Fujian, China; 2 Xiamen Neurosurgical Quality Control Center, Xiamen, Fujian, China; 3 National Institute for Data Science in Health and Medicine, Xiamen University, Xiamen, Fujian, China; 4 Department of Neurosurgery and Department of Neuroscience, Fujian Key Laboratory of Brain Tumors Diagnosis and Precision Treatment, The First Affiliated Hospital of Xiamen University, School of Medicine, Xiamen University, Xiamen, Fujian, China

**Keywords:** digital neurosurgery, 3D imaging, 3D printing, digital twins, artificial intelligence, robotic surgery, surgical navigation, neurosurgery technologies

## Abstract

**Background:**

Digital neurosurgery represents a transformative shift in modern neurosurgical practice, integrating advanced technologies, such as three-dimensional (3D) imaging, robotics, artificial intelligence (AI), and digital twin technology (DTT) models. These technologies offer innovative solutions for preoperative planning, intraoperative navigation, and postoperative management, with an emphasis on precision, personalization, and efficiency.

**Methods:**

We conducted a scoping review in accordance with the Preferred Reporting Items for Systematic Reviews and Meta-Analyses-Scoping Review (PRISMA-ScR) checklist and guidance from the Joanna Briggs Institute (JBI) Manual for Evidence Synthesis. PubMed, Web of Science (WOS), and China National Knowledge Infrastructure (CNKI) were searched without language or date limits through September 2025. Eligibility was structured using the Population–Concept–Context (PCC) framework. Two reviewers independently screened records in Rayyan with consensus resolution, and data were charted using a prepiloted form. A total of 133 sources were included and mapped.

**Results:**

Key technologies reviewed include: (1) 3D reconstruction: Facilitates precise anatomical modeling, improving spatial understanding and surgical planning. (2) 3D printing (3DP): Enables creation of patient-specific models and surgical guides, enhancing preoperative simulation and intraoperative accuracy. (3) Digital twins (DT): Offers dynamic virtual models for real-time surgical simulation, training, and personalized patient management. (4) Intraoperative navigation: Utilizes advanced electromagnetic and AI-enhanced systems to improve tracking accuracy and reduce surgical errors. (5) Robotic-assisted surgery: Includes telesurgical, supervisory, and handheld systems that enhance precision and enable minimally invasive procedures. (6) AI: Supports image registration, subtask automation, and clinical decision-making, improving diagnostic and prognostic accuracy. These technologies demonstrate significant benefits in operative precision, patient outcomes, training efficacy, and interdisciplinary communication, though challenges remain in data integration, regulatory standards, and computational demands.

**Conclusion:**

Positioning this study as a scoping review clarifies its objective to map technologies and applications across digital neurosurgery rather than to synthesize effect estimates, thereby providing an evidence-informed overview to guide future systematic evaluations. Digital neurosurgery is rapidly evolving toward greater integration of multimodal data, real-time adaptive systems, and AI-driven automation. Future developments should focus on standardizing regulatory frameworks, enhancing data fusion capabilities, and promoting interdisciplinary collaboration to fully realize the potential of digital technologies in advancing neurosurgical care.

## 1 Introduction

Digital neurosurgery is at the forefront of modern neurosurgical development. It is not a standalone surgical procedure, but rather a treatment model that integrates individualized patient data at its core, deeply incorporating medical imaging, computer technology, DT, artificial intelligence, and other digital technologies. This model transforms the entire process of preoperative planning, intraoperative navigation, postoperative evaluation, and patient rehabilitation for neurosurgical diseases. The critical technological components and application areas involve highly personalized, accurate preoperative planning, real-time intelligent intraoperative navigation systems, efficient and scientific postoperative management and follow-up, an extensive training and teaching framework, and a visual and intuitive model for doctor-patient communication. After several years of rapid progress, digital neurosurgery technologies have been extensively utilized in clinical neurosurgical practice, encompassing subspecialties like trauma neurosurgery, brain tumor surgery, functional neurosurgery, vascular neurosurgery, and spinal surgery.

Despite the challenges in the development of digital neurosurgery, such as the requirement for skilled operators, high costs of certain equipment, and the complexity of establishing a digital ecosystem across different devices, it is undeniable that digital neurosurgery has significantly advanced the neurosurgery field. The digital revolution has fundamentally changed the neurosurgery profession. At present, while digital neurosurgery is rapidly advancing, issues have arisen, such as a lack of information sharing between clinicians and engineers in the computer vision field, redundant technical research or unclear research directions, and mismatches between different technologies and various clinical scenarios.

Accordingly, we aimed to map (rather than pool) the breadth of digital technologies in neurosurgery, describe their clinical use cases across the perioperative continuum, and identify gaps for future hypothesis-driven systematic reviews and comparative studies. We therefore designed this study as a scoping review. This scoping review focuses on several key technologies in the current digital neurosurgery field, assessing their clinical effectiveness and development potential. It aims to help clinical professionals and researchers quickly and thoroughly understand these technologies, inspire breakthroughs in future research, and offer ideas for the future progression of digital neurosurgery.

## 2 Methods

We conducted a scoping review following the PRISMA-ScR checklist (1) and the JBI Manual for Evidence Synthesis (2) (Supplementary File 1). The review protocol was developed *a priori*; no meta-analysis or study-level risk-of-bias assessment was planned, consistent with scoping methodology.

### 2.1 Article collection

This review’s data were obtained from relevant articles published on PubMed,^1^ WOS,^2^ and CNKI.^3^ There were no language restrictions for the articles retrieved. Researchers used Medical Subject Headings (MeSH) terms for keyword searches, with no restrictions on publication dates, and the search was conducted until September 2025. EndNote 21 (version 21.4.0) was used to remove duplicate literature. The Rayyan web application (Professional version: https://www.rayyan.ai/) was employed to ensure a blinded screening process. A detailed search strategy is provided in Table 1.

**Table 1:** Detailed search strategy.

Concept block (combined with AND)	MeSH terms (combined with OR)	Title/abstract terms (combined with OR)
Neurosurgery domain	“Neurosurgical Procedures”[MeSH] OR “Neurosurgery”[MeSH] OR “Brain Diseases/surgery”[MeSH] OR “Spinal Diseases/surgery”[MeSH]	Neurosurg*[Title/Abstract] OR “Neurosurgical procedure*”[Title/Abstract] OR “Brain surg*”[Title/Abstract] OR “Spine surg*”[Title/Abstract]
Technologies	“Three-Dimensional Imaging”[MeSH] OR “Printing, Three-Dimensional”[MeSH] OR “Surgical Navigation Systems”[MeSH] OR “Robotics”[MeSH] OR “Robot-Assisted Surgery”[MeSH] OR “Artificial Intelligence”[MeSH] OR “Machine Learning”[MeSH] OR “Deep Learning”[MeSH]	“3D imaging”[Title/Abstract] OR “3D reconstruction”[Title/Abstract] OR “Three dimensional Reconstruction”[Title/Abstract] OR “3D printing”[Title/Abstract] OR “Three dimensional printing”[Title/Abstract] OR “Digital twin”[Title/Abstract] OR “Virtual patient”[Title/Abstract] OR “Virtual model”[Title/Abstract] OR “Surgical navigation”[Title/Abstract] OR “Intraoperative navigation”[Title/Abstract] OR “Robot assisted”[Title/Abstract] OR “Robotics”[Title/Abstract] OR “Artificial intelligence”[Title/Abstract] OR “AI”[Title/Abstract] OR “Machine learning”[Title/Abstract] OR “Deep learning”[Title/Abstract]
Outcomes/use cases	“Diagnosis”[MeSH] OR “Therapy”[MeSH] OR “Treatment Outcome”[MeSH] OR “Education, Medical”[MeSH] OR “Physician-Patient Relations”[MeSH] OR “Communication”[MeSH]	Diagnos*[Title/Abstract] OR Therap*[Title/Abstract] OR Treat*[Title/Abstract] OR Outcome*[Title/Abstract] OR Educat*[Title/Abstract] OR Teach*[Title/Abstract] OR Training[Title/Abstract] OR “Physician patient relation*”[Title/Abstract] OR “Doctor patient Communication”[Title/Abstract] OR communicat*[Title/Abstract]

Within each cell, terms are combined with OR. The three concept blocks are combined with AND.

### 2.2 Inclusion criteria and exclusion criteria

Inclusion criteria: studies were considered eligible if they met the following criteria: (1) the study population included patients undergoing neurosurgical procedures (e.g., brain or spinal surgery) or settings related to neurosurgical practice, including education and physician–patient communication; (2) the intervention involved digital-assisted technologies, such as 3D reconstruction, 3D printing, DT models, intraoperative navigation systems, robot-assisted surgery, or artificial intelligence (including machine learning and deep learning); (3) the study reported at least one relevant outcome, such as diagnostic accuracy, therapeutic effect or treatment outcome, medical education/training, or physician–patient communication; (4) the study design was an original investigation, including randomized controlled trials, cohort studies, case–control studies, cross-sectional studies, systematic reviews/meta-analyses, or case series; and (5) the articles were published in peer-reviewed journals.

Exclusion criteria: Studies were excluded if they met any of the following criteria: (1) not related to neurosurgery (e.g., studies in cardiothoracic, orthopedic, or other surgical specialties); (2) animal research or carcass research; (3) did not involve digital-assisted technologies, focusing solely on conventional surgical or imaging methods; (4) did not report outcomes related to diagnosis, therapy, education, or communication; (5) were non-original publications, including conference abstracts, editorials, expert opinions, letters, book chapters, or non-peer-reviewed materials; and (6) duplicate reports of the same study.

### 2.3 Article screening process

First, a preliminary screening of the collected articles and their associated Medical Subject Headings (MeSH) terms was conducted based on the inclusion and exclusion criteria. Next, the studies were filtered by article title, and those with titles that did not match the criteria were excluded. Finally, the remaining articles were further screened based on their data, selecting those that contained the required information for this study while excluding others. The two researchers (ZY and ZS) screened the titles and abstracts, and any disagreements were resolved through discussion to reach a consensus.

### 2.4 Data retrieval

The researchers manually screened the collected articles and extracted the required data. The data extracted includes literature type, publication year, PubMed ID, types of neurological diseases or surgeries, study population, the digital neurosurgery technologies used in the study, traditional techniques, and the study outcomes, i.e., the impact of novel treatment methods on patients as compared to conventional methods. The extracted data were organized and analyzed in accordance with the adjusted PCC framework (3) (Table 2). No quantitative pooling or formal certainty grading was attempted.

**Table 2:** PCC framework for digital neurosurgery.

Element	Operational definition for this review	Illustrative examples/keywords
Population	Patients undergoing neurosurgical care (cranial/spinal), as well as settings involving neurosurgical training or clinician–patient communication.	Brain/spine surgery patients; neurosurgical trainees; preoperative counseling.
Concept	Digital technologies relevant to neurosurgery.	3D reconstruction; 3D printing; digital twin/digital twin technology; intraoperative navigation; robotics; artificial intelligence.
Context	Perioperative contexts spanning diagnosis, planning, intraoperative guidance, postoperative management, education, and communication.	Diagnostic work‑up; presurgical planning; intraoperative guidance; postoperative follow‑up; education; communication.

Items within cells are listed as provided.

## 3 Results

**Figure 1:**
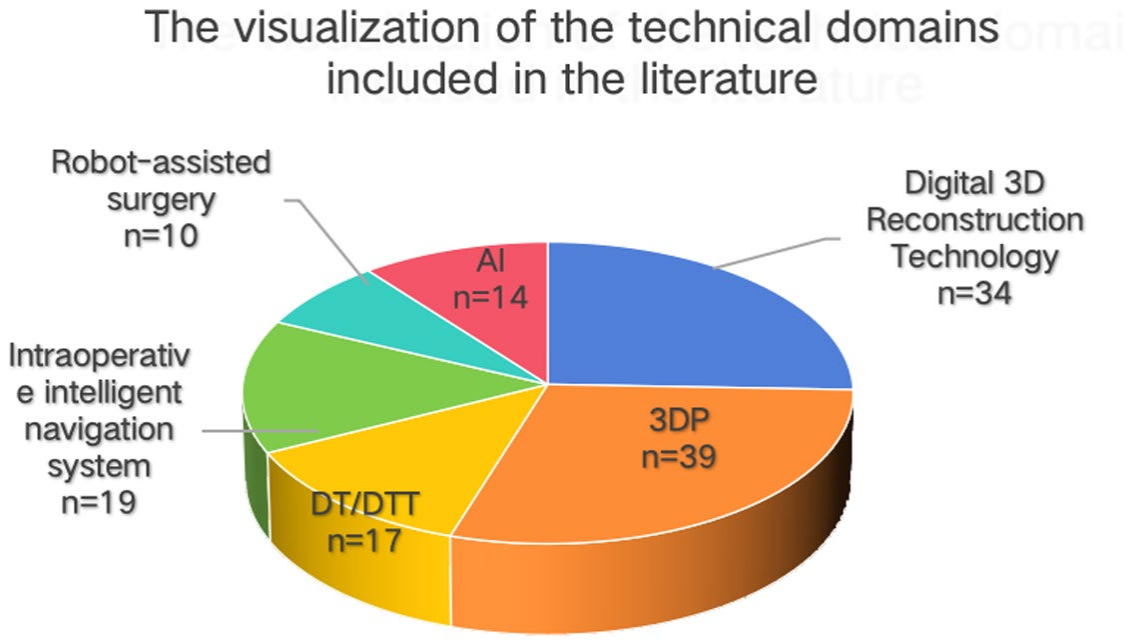
Distribution of included studies across digital-neurosurgery technology domains. This pie chart summarizes how the 133 included sources were mapped to six domains: 3D printing (3DP, *n* = 39; 29.3%), digital 3D reconstruction technology (*n* = 34; 25.6%), intraoperative intelligent navigation systems (*n* = 19; 14.3%), digital twins/digital-twin technology (DT/DTT, *n* = 17; 12.8%), artificial intelligence (AI, *n* = 14; 10.5%), and robot-assisted surgery (*n* = 10; 7.5%). Wedge sizes are proportional to counts, which sum to 133.

**Figure 2:**
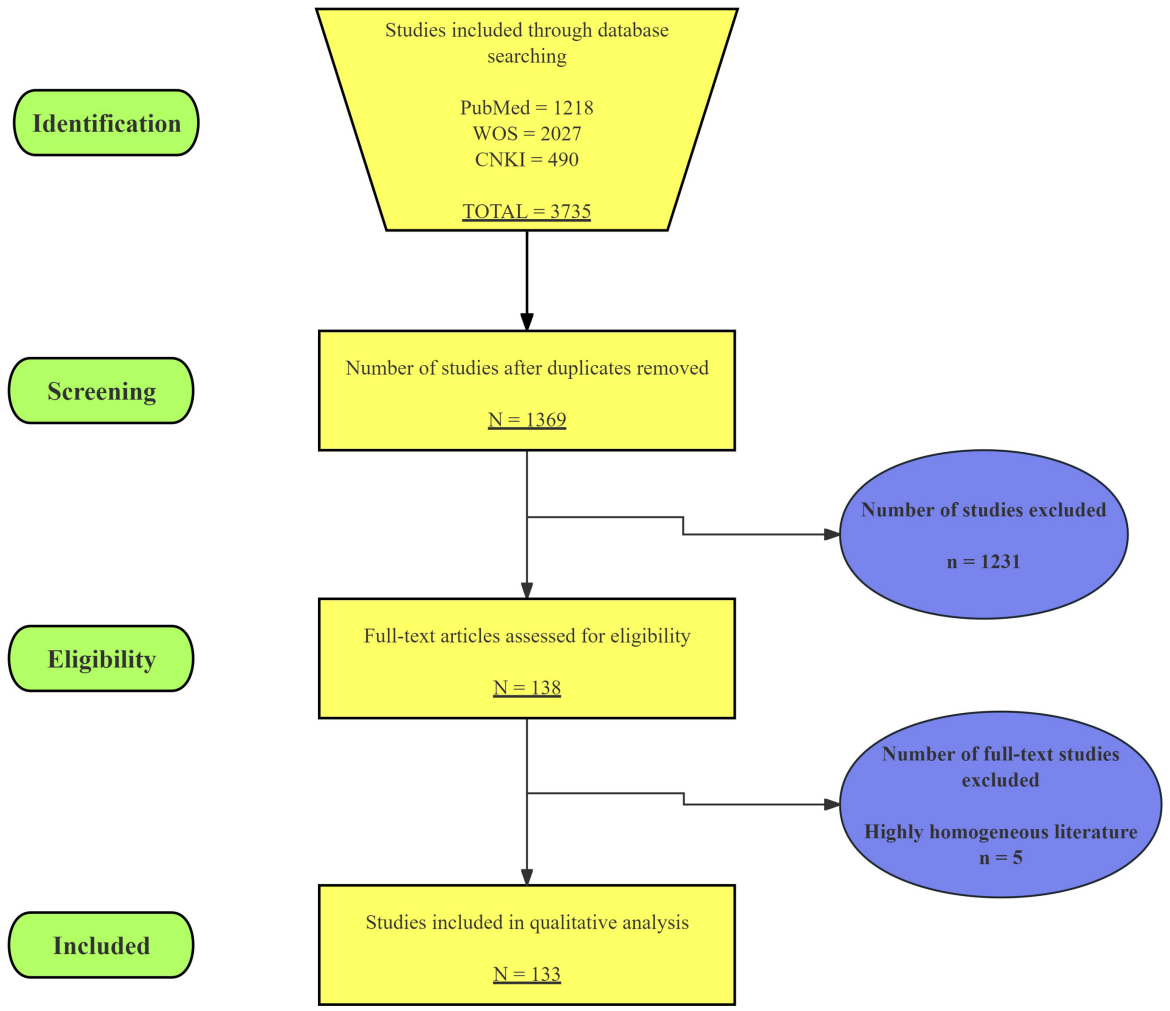
PRISMA-ScR flow diagram of study selection.

The initial search retrieved a total of 3,735 articles, with 1,218 from PubMed, 2,027 from WOS, and 490 from CNKI. After deduplication, 1,369 articles remained. Two researchers independently screened the titles and abstracts, excluding 1,231 articles that did not meet the criteria. The two researchers then conducted full-text eligibility assessments on the remaining 138 articles, excluding 5 due to high homogeneity and other reasons. Record import, deduplication, and blinded screening were performed with the assistance of the web-based application Rayyan; all outputs were independently and manually verified by two reviewers. In the end, 133 sources were charted (Figure 1). The screening process is shown in Figure 2. The summary of key auxiliary technologies related to digital neurosurgery discussed in the results is presented in Table 3.

**Table 3:** Summary of widely used technologies in digital neurosurgery.

Technology	Method description	Three most important publications	Clinical applications in daily routine	Research institutes where development is performed	Number of patients enrolled in clinical studies/experiments
Digital 3D reconstruction technology	Builds precise anatomical 3D models from imaging to improve spatial understanding and surgical planning.	Karako et al. (3); Verykokou and Ioannidis (4); Ingale and DU (8).	Used for pre-operative planning and precise anatomical modeling in clinical workflows.	Graduate School of Engineering, Chiba University (3). Laboratory of Photogrammetry, School of Rural, Surveying and Geoinformatics Engineering, National Technical University of Athens (4). School of Information Technology and Engineering, Vellore Institute of Technology (8).	Data base Scopus, IEEE Xplore, Science Direct, ACM Digital Library and Springer Link (8).
3DP	Creates patient-specific models and surgical guides; supports presurgical simulation and intraoperative accuracy.	Pucci et al. (14); Klein et al. (32); Thawani et al. (33).	Presurgical models, intraoperative guides, and implant-related uses are described.	Columbia University Medical Center Department of Neurological Surgery (14) Departments of Neurological Surgery & Rehabilitation Medicine, University of Miami Miller School of Medicine (32) Department of Neurosurgery, Univer-sity of Pennsylvania (33).	Three patient DLGGs (33).
DT & DTT	Interactive digital models supporting pre-op planning, intra-op situational awareness/decision-making, postoperative data capture, and education/training.	Kamel Boulos and Zhang (39); Wang et al. (42); Fekonja et al. (43).	Reported across brain and spinal surgeries; training and peri-operative assessment roles highlighted; large-scale routine use not established in the manuscript.	Information Management School, Sun Yat-sen University (39). Aix Marseille Université, Institut National de la Santé et de la Recherche Médicale, Institut de Neurosciences des Systèmes (INS) (42). Department of Neurosurgery, Charité - Universitätsmedizin Berlin (43).	Retrospective evaluation (VEP): *n* = 53 drug-resistant focal epilepsy patients (42). Prospective clinical trial (EPINOV, NCT03643016): planned/enrolled *n* = 356 epilepsy patients (42).
Intraoperative intelligent navigation system	Traditional stereotactic navigation (3D coordinates) is long-standing and mainstream; MRI (fast sequences) and MRI-guided robots provide precise real-time guidance; electromagnetic navigation improves accuracy/safety.	Guo et al. (60); Uecker et al. (61); Harwick et al. (62).	Described as mainstream with standardized procedures; MRI/EM enhancements applied in practice.	Department of Mechanical Engineering, The University of Hong Kong (60). Biomedizinische NMR Forschungs GmbH am Max-Planck-Institut für biophysikalische Chemie (61). Department of Neurosurgery, Medical College of Wisconsin and Froedtert Hospital (62).	DBS with ClearPoint *n* = 27 adults; PD with ClearPoint *n* = 26; NeuroArm series *n* = 56 and another *n* = 22; NeuroBlate multicenter LITT *n* = 34 (24 GBM + 10 anaplastic glioma) (60). Eight subjects for speech production and 12 for cardiac imaging (some scanned repeatedly) (61). Ten awake pinless intra-axial tumor resections using Curve EM neuronavigation (62).
Robot-assisted surgery	Three categories: telesurgical (e.g., NeuroArm), supervisory surgeon-controlled (e.g., SpineAssist/Renaissance), and handheld shared-control (e.g., Steady-Hand; NeuRobot). Widely applied in spinal surgery; one platform reported in >1,000 neurosurgical procedures.	Bagga and Bhattacharyya (73); Overley et al. (77); Menaker et al. (76).	Routine in spine workflows; devices noted/approved for brain use; >1,000 procedures reported for NeuroArm.	Sheffield Teaching Hospitals (73). The Mount Sinai Hospital, Icahn School of Medicine (77). Department of Neurological Surgery, University of Miami Miller School of Medicine (76).	Fifteen patients for robotic cerebral angiography; 501 neurosurgical procedures with a robotic DSA system; a 20-patient cohort using an auto-navigating microscope (76).
AI	Supports image registration, sub-task automation, path planning, and decision support; evidence base in neurosurgery is growing.	Han et al. (86); Senders et al. (95); Schonfeld et al. (87).	Emerging clinical use; systematic review suggests ML complements expert work; broader, large-scale application not yet established.	Johns Hopkins Medicine (Neurosurgery and Radiology) (86). Department of Neurosurgery, University Medical Center (95). Neurosurgery, Stanford University School of Medicine (87).	Nine longitudinal MR/CT subjects with real brain deformation and 16 neurosurgery cases (preop MR + intra/postop CT) in an IRB-approved retrospective clinical study (86).

All entries strictly reflect the manuscript content and its reference list; 3DP, 3D print; DT, digital twin; DTT, digital twin technology; AI, artificial intelligence.

Across the 133 included studies, evidence clustered into six domains (3D reconstruction/printing, DT/DTT, navigation, robotics, AI). The majority of reports were single-center technical/observational studies; randomized comparisons were rare. Consistent directional benefits included improved anatomic understanding and planning fidelity (3D), guide/trajectory accuracy (3DP), tracking precision and brain-shift mitigation (navigation with intra-op imaging), stability in constrained corridors (robotics), and registration/decision support (AI). However, heterogeneity and a predominance of surrogate engineering metrics temper certainty regarding patient-centered outcomes.

### 3.1 Digital 3D reconstruction technology

Digital 3D reconstruction technology is among the most prominent research fields in computer vision. This technology relies on computer technology and digital methods to convert two-dimensional data containing object or structural information into three-dimensional models in visual space. The core of the technology involves multiple disciplines, including image processing, stereoscopic vision, and others, aiming to transform real-world scenes (objects) into mathematical models compatible with computer formats (4–6). The advantages of digital 3D reconstruction technology include digital storage, 3D visualization, accurate reproduction, and the reconstruction of digital models (7).

In digital neurosurgery, digital 3D reconstruction technology is a critical core technology, and the 3D models it reconstructs are vital for clinicians in diagnosing and treating patients. Digital 3D reconstruction technology can capture, reproduce, process, analyze, and understand both static and dynamic images of neurosurgical procedures in real-time (6, 8), which means it can better diagnose various diseases and enable deeper analysis. When neurosurgeons use the digital models reconstructed in 3D, the advantages of the spatial model are primarily reflected in:

1. The 3D digital model based on the patient’s true condition is both realistic and accurate. While ensuring high precision, it can also be professionally processed in real-time, maximizing the satisfaction of clinical needs across different departments.
2. The 3D digital model compensates for the loss of depth information, shape distortion, and difficulty in accurately understanding spatial relationships that are common in a single perspective on a two-dimensional plane, providing a comprehensive experience for doctors, patients, and researchers (9). It also provides spatial information such as the structural depth of the target object and positional adjacency relationships, which cannot be offered by two-dimensional plane images (10).
3. The 3D digital model has greater interactivity. Doctors or researchers can operate and modify the digital model using wired or wireless signal transmission (LAN or Wi-Fi), allowing them to interactively observe the target from any angle according to their needs. Furthermore, the digital imaging model of the 3D spatial structure can rely on 5G high-speed network signals for cross-regional, delay-free transmission, ensuring the highest efficiency and accuracy in communication across regions (11, 12).

In theory, the 3D digital model can be stored indefinitely in digital format (13). The digital models are usually paired with specialized software designed for the accurate and reliable analysis of 3D data. Convenient access methods significantly reduce the pressure on physical storage space, and regional expertise can be shared globally with different experts via the internet.

**Figure 3:**
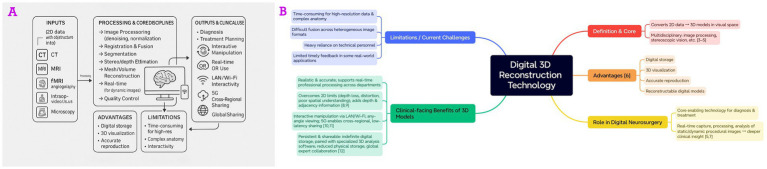
Application framework of digital 3D reconstruction. **(A)** Workflow converting multimodal 2D images into patient-specific 3D models for diagnosis, planning, interactive/operating room use, with key advantages and limitations indicated. **(B)** Mind map summarizing definition, advantages, role, clinical benefits, and current challenges.

Due to these characteristics, digital 3D reconstruction technology is regarded as the cornerstone of digital neurosurgery. Although current reconstruction technology still exposes many issues, such as being more time-consuming when processing high-resolution data and complex anatomical structures, difficulties in merging image data from different formats, excessive reliance on technical personnel, and the lack of timely feedback from digital models in certain real-world applications (Figure 3).

### 3.2 3DP

3D printing is an additive manufacturing (AM) method, where, after computer-aided design (CAD), a 3D printer constructs a three-dimensional structure layer by layer in a specific sequence (14). 3DP can spatially reproduce digital models in real-world environments with precise dimensions, a feature that makes this technology highly attractive in surgical procedures (15). Recent developments in 3DP technology, including cost reductions and more advanced computer engineering, have facilitated its widespread adoption in various application scenarios (16). For many years, 3D printing technology has offered various solutions for clinicians in the surgical field, thanks to its unique features: (1) 3DP used as a presurgical tool. (2) 3DP used as intrasurgical tool, and (3) 3DP used as an implant or replacement (14).

As a presurgical tool, 3DP offers surgeons a simple but powerful solution: the physical 3DP model. Based on real patient data, such as lesion location and anatomical structure, clinicians can use digital 3D reconstruction technology to create detailed models and process them according to clinical needs, ultimately producing a virtual model with high precision (17). Surgeons can use these virtual models for preoperative planning because the creation of high-precision models enables clinicians to envision potential scenarios that may occur when opening the patient’s body during surgery (18). Furthermore, the presurgical 3DP models can fulfill higher personalization demands and more targeted preoperative design requirements, whether for substitutes or assistive tools used during surgery (19, 20). Better planning solutions, more intuitive visualization effects, safer surgeries, and lower costs are the clinical benefits of using 3DP anatomical models (21).

As an intraoperative tool, 3DP enables clinicians and engineers to produce customized auxiliary tools for use during surgery. One of the more mature auxiliary tools is the 3DP surgical guide (22, 23). A surgical guide is an intraoperative navigation tool created using a process similar to that of anatomical models. The surgical guide simulates the complex surfaces of the human body or organs to create a fixture that enables the surgeon to perform precise cutting, drilling, or excision during surgery. Compared to traditional surgery without auxiliary tools, the use of a guide made with 3DP has been proven effective in excisional surgeries (23, 24).

At present, researchers are primarily focused on how to use 3DP as a functional implant, and how it could serve as a solution for replacing entire organs in the future (25, 26). Digital 3D reconstruction technology can use imaging data from real organs to accurately reconstruct anatomical models, which has sparked discussions over a long period about whether these “manufactured products” could evolve into useful organs (27). Scientists have begun designing 3DP scaffolds with complex structures, and it has been proven that these scaffolds serve as an excellent medium for promoting cell activity and differentiation (28). 3DP has now been successfully applied in surgeries such as orthopedics, dentistry, maxillofacial surgery, and neurosurgery, where 3DP is used to create implants with complex shapes and topological structures to meet personalized surgical needs (29, 30).

Moreover, 3DP technology plays an essential role in neurosurgical medical education. The rapid application of 3DP technology in surgical training is partly due to the increasing demand for proficiency in surgical skills in environments with limited working hours (31). One reason is the complexity of surgical practice, which has resulted in the use of human models during the early training stages of various surgical residents, including those in neurosurgery (32, 33). The resulting surgical simulation devices serve as a reasonable supplement during the internship phase. According to the literature, models simulating neurosurgical vascular and skull base techniques are relatively common. Another reason is that 3DP models simulating epilepsy surgery and brain tumor microanatomy techniques are less frequently described in the literature (34–36). Therefore, we believe that 3DP technology has not yet developed to the stage where it can create models capable of simulating fine anatomical details.

**Figure 4:**
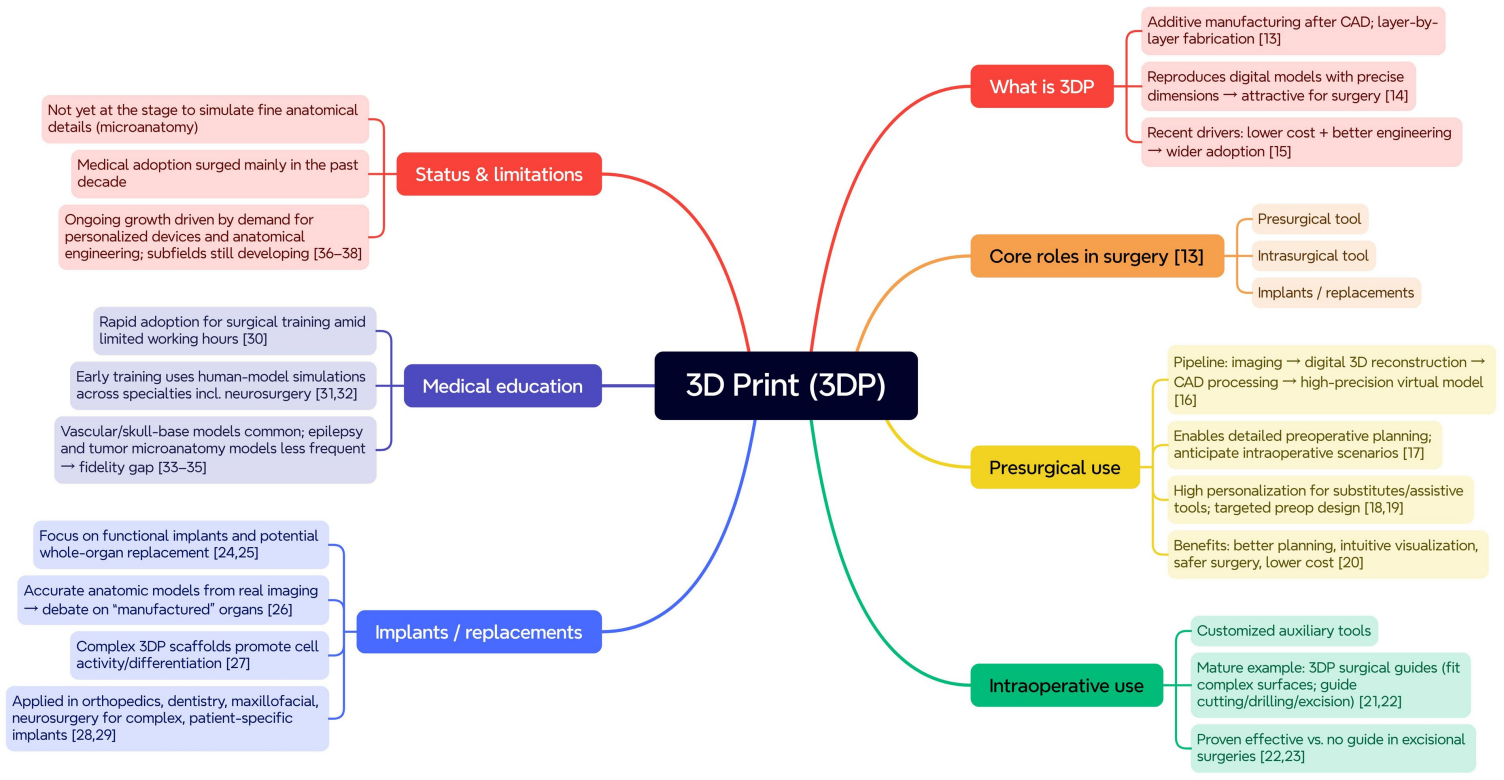
Application framework of 3DP. Mind-map showing: definition, roles (pre-/intra-operative tools; implants), uses (planning pipeline and surgical guides), education, and current status/limitations (rapid adoption yet limited micro-anatomical fidelity).

3DP is not a novel technology, but it has only begun to be widely applied in the medical field over the past decade (Figure 4). As the demand for personalized assistive devices and anatomical engineering grows among patients, doctors, and researchers, applications in the related subfields of 3DP are still being actively developed (37–39).

### 3.3 DT and DTT

**Figure 5:**
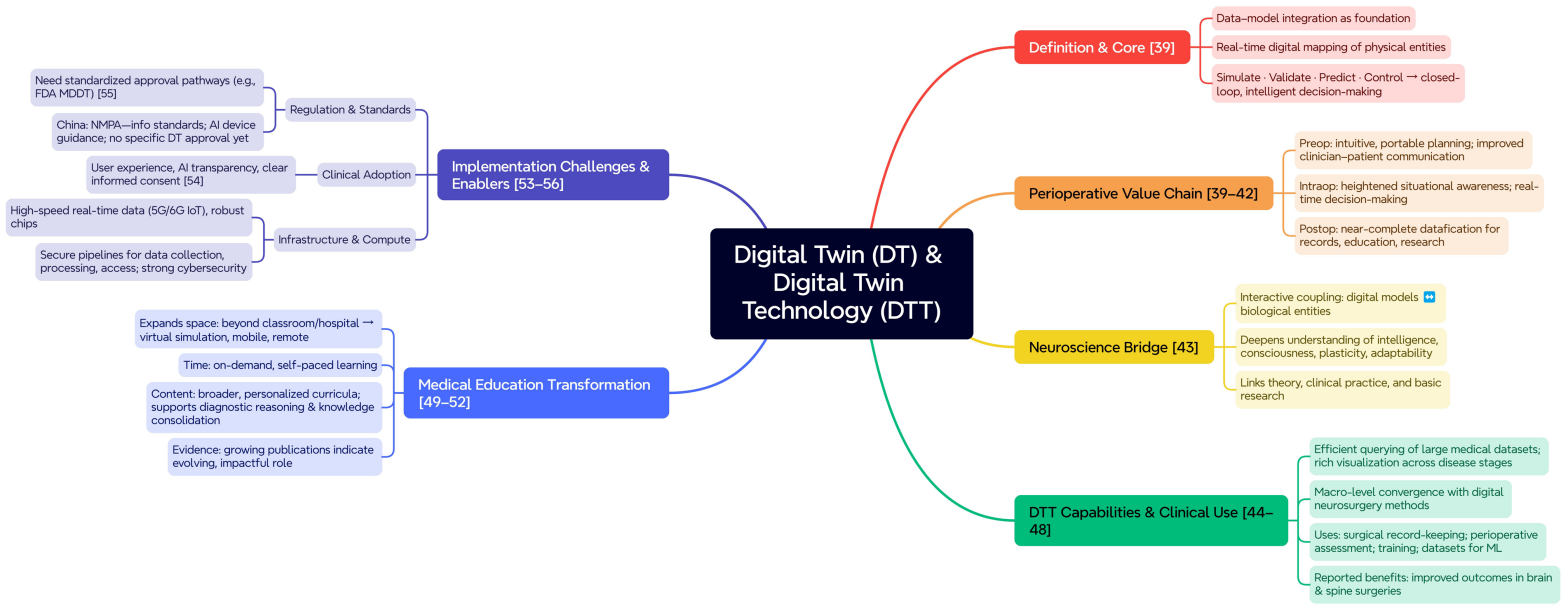
Application framework of DT and DTT. Mind-map of DT/DTT’s core, perioperative value (pre/intra/post), neuroscience bridge, capabilities/uses, implementation challenges/enablers (regulation, adoption, infrastructure/cybersecurity), and education transformation.

DT is a digital concept and technology that relies on the integration of data and models as its foundation and core. It creates accurate digital mappings of physical objects in real-time within a digital space, and uses data integration and predictive analysis to simulate, validate, predict, and control the entire lifecycle of physical entities, ultimately forming an optimized closed-loop for intelligent decision-making (40) (Figure 5).

DT utilizes the highly digitalized and sensor-laden medical environment of today to construct virtual models of patients’ neuroanatomical structures and clinical characteristics, enhancing the quality of care in perioperative management (41, 42). Preoperatively, clinicians can use DT-related technologies for more intuitive and portable surgical planning, enhancing efficiency and improving doctor-patient communication with a more humanistic approach. Intraoperatively, the surgeon can enhance situational awareness of the affected areas and real-time decision-making ability using DT-related technologies. Postoperatively, DT-related technologies can enable nearly complete digitization of all types of patient data throughout the perioperative period for record-keeping and facilitating subsequent education and research (40, 43). In neuroscience, the advantage of DT is reflected in the interaction between digital models and biological entities. Their adaptability not only deepens our understanding of complex physiological phenomena but also enables us to reconsider the theoretical concepts behind phenomena like intelligence, consciousness, plasticity, and adaptability. Therefore, DT also holds the potential to bridge the gap between theoretical research, clinical practice, and basic research in the field of neuroscience (44).

The integration of DTT allows medical teams to efficiently query and utilize large medical datasets, enabling comprehensive reproduction of a patient’s condition at different stages of the disease using various visualization techniques (45). At present, most surgical methods in digital neurosurgery can be converged and integrated, making it easier to combine them with DTT at a macro level. Currently, DTT can enhance the operator’s situational awareness of the overall surgical environment to varying degrees, and it is used for surgical record-keeping, comprehensive perioperative assessment, surgical training, and generating real datasets for machine learning algorithm development. Many studies have shown that these methods have been applied in various brain and spinal surgeries, resulting in better surgical outcomes (46–49). In addition, DTT offers transformative opportunities for improving the training levels of medical professionals. In the past, Friedman believed that medical education in the United States was in a “crisis” across 3D: physical space, time, and content (50). The emergence and widespread use of DTT have significantly expanded the spatial dimension, extending beyond classrooms and hospitals to virtual simulations, mobile learning, and remote sites, making learning environments more flexible. In terms of time, on-demand learning and self-controlled learning pace have become the norm in medical education. Additionally, the growth in content dimensions showcases a broad range of topics that can be tailored to individual learner needs. Beyond skill training, the use of DTT in the teaching and training of various medical specialties has grown, helping to develop diagnostic reasoning skills and solidify professional knowledge (51, 52). Overall, the growing number of publications over the years shows that DTT is evolving and playing an important role in transforming and overcoming the traditional constraints of medical education (53).

The challenges faced in the implementation of DTT have been divided into several key areas: regulatory standards, clinical applications, and computational requirements (54, 55). Currently, in response to regulatory issues, a key regulatory step is the establishment of a standardized approval process by national medical and drug regulatory agencies (such as the U.S. Food and Drug Administration, FDA) that aligns with the interests of each country. Despite the extensive publicly available research data proving the effectiveness and safety of DTT in clinical applications, the heterogeneity and lack of transparency in DTT have obstructed the regulatory processes for specific technologies. In response, the FDA initiated the Medical Device Development Tools (MDDT) program (56). In China, the National Medical Products Administration (NMPA) has not established an independent approval process or released specific technical guidelines for “DTT.” DTT, as an emerging digital technology, involves multiple fields such as medical devices and software products. In 2023, the NMPA released the “Pharmaceutical Regulatory Information Standards System,” which covers content such as software development process specifications and applies to the planning, requirements, design, development, integration, testing, and other processes of pharmaceutical regulatory informatization. In addition, for artificial intelligence medical devices, the NMPA has also issued relevant guidance documents, such as the “Guidelines for the Registration and Review of AI Medical Devices (Draft for Comments),” which outlines general requirements for the lifecycle management of AI medical devices and introduces registration document requirements.

The clinical application of DTT requires the support of both clinicians and patients. This support will be based on optimizing user experience, improving the transparency of AI models, and having a clear informed consent process (55). The hardware requirements, such as computer chips and network signal base stations, may ultimately become the biggest challenges faced during the large-scale implementation of DTT. A key requirement is the widespread development of infrastructure capable of high-speed real-time data transmission (i.e., 5G and 6G bandwidth Internet of Things). In the healthcare sector, these high-speed data transmission facilities will be crucial for the real-time integration of patient medical data into DT models. Currently, to support the collection, processing, and access of medical data that underpin DTT, powerful computing capabilities and robust network security measures are essential (57).

### 3.4 Intraoperative intelligent navigation system

Traditional stereotactic neurosurgical navigation technology is a method that uses a 3D coordinate system to pinpoint target sites within the brain. Essentially, this technology is an auxiliary surgical method that associates the position of instruments in the surgical field with anatomical structures in 3D space (58). This approach has been used in the field of neurosurgery for several decades. The inherent accuracy of traditional stereotactic navigation technology has made it possible to precisely treat functional disorders, epilepsy, and brain tumors through surgery and radiation therapy (58). Although traditional stereotactic navigation technology is still considered a mainstream navigation method and widely used, with standardized surgical procedures established over decades, stereotactic surgery remains challenging, especially with the high demands for surgical precision and minimal invasiveness. Inaccurate instrument positioning can cause trajectory deviations and targeting errors, which significantly elevate the risk of bleeding. Therefore, in current surgical procedures, clinicians and researchers have adopted various strategies to reduce errors (59, 60). However, without real-time updates of the instrument’s position relative to the target, these strategies cannot compensate for the continuously changing circumstances during surgery. The registration during the planning phase (image fusion) is likely to be based solely on the patient’s preoperative imaging data as the sole reference for surgical route planning (61). Thus, the reliance solely on preoperative imaging data to plan the surgical route appears to be a major limitation of traditional stereotactic navigation technology. As a result, given the current challenges in neurosurgery, advancements in real-time visualization and high-precision operations are vital for optimizing intraoperative navigation systems and streamlining procedural workflows. Compared to other imaging methods (such as ultrasound or CT), MRI offers several advantages in intraoperative guidance, as it is highly sensitive to intracranial pathological/physiological changes and can display soft tissues with high-contrast images without ionizing radiation. Fast MRI sequences (such as radial fast imaging using low-angle shot sequence with a temporal resolution of 20–30 ms) (62) have been widely applied in MRI equipment and can now be used for surgical guidance when soft tissue deformation is involved. Currently, MRI-guided robots are being applied in more complex neurosurgical procedures, providing precise stereotactic guidance for image-guided treatments, such as device implantation or tissue ablation (61), which seems to be a major upgrade to traditional navigation systems.

With the integration of medical imaging technology and electronic information technology, electromagnetic navigation technology has played a crucial role in improving the accuracy and safety of neurosurgical procedures (63). Electromagnetic navigation technology is an advanced surgical assistance system that integrates a 3D navigation system with a magnetic field sensing device to enable the precise positioning of surgical instruments inside the patient’s body. By combining preacquired image data with real-time electromagnetic field information, surgical instruments can be accurately tracked in three-dimensional space and navigated to the target location, thereby improving surgical precision and reducing the risk of damage (64). The application of electromagnetic navigation technology in neurosurgery for spinal and cranial surgeries has been proven to significantly improve surgical outcomes, including increasing the resection rate of deep brain tumors, the accuracy of pedicle screw implantation, and reducing intraoperative bleeding (65, 66). Currently, researchers and neurosurgeons have introduced machine vision-based correction algorithms to monitor and correct magnetic field changes caused by surgical actions in real-time, ensuring the real-time reliability of electromagnetic navigation data (67). On this basis, researchers have significantly enhanced the efficiency and accuracy of the electromagnetic navigation system in handling complex clinical image data by integrating multimodal imaging data, such as real-time registration of existing preoperative CT and MR images with intraoperative ultrasound images. Particularly, deep learning and machine learning algorithms perform rapid image processing and pattern recognition, ensuring fast and accurate registration during the use of the electromagnetic navigation system (68). In subsequent research, a team led by clinicians optimized the materials of existing electromagnetic navigation devices, reducing weight while ensuring the integrity of the equipment. Additionally, a modular design approach was employed to enhance maintainability and upgradeability. These comprehensive measures have greatly enhanced the user experience and versatility of the current portable neuroelectromagnetic navigation devices (69).

**Figure 6:**
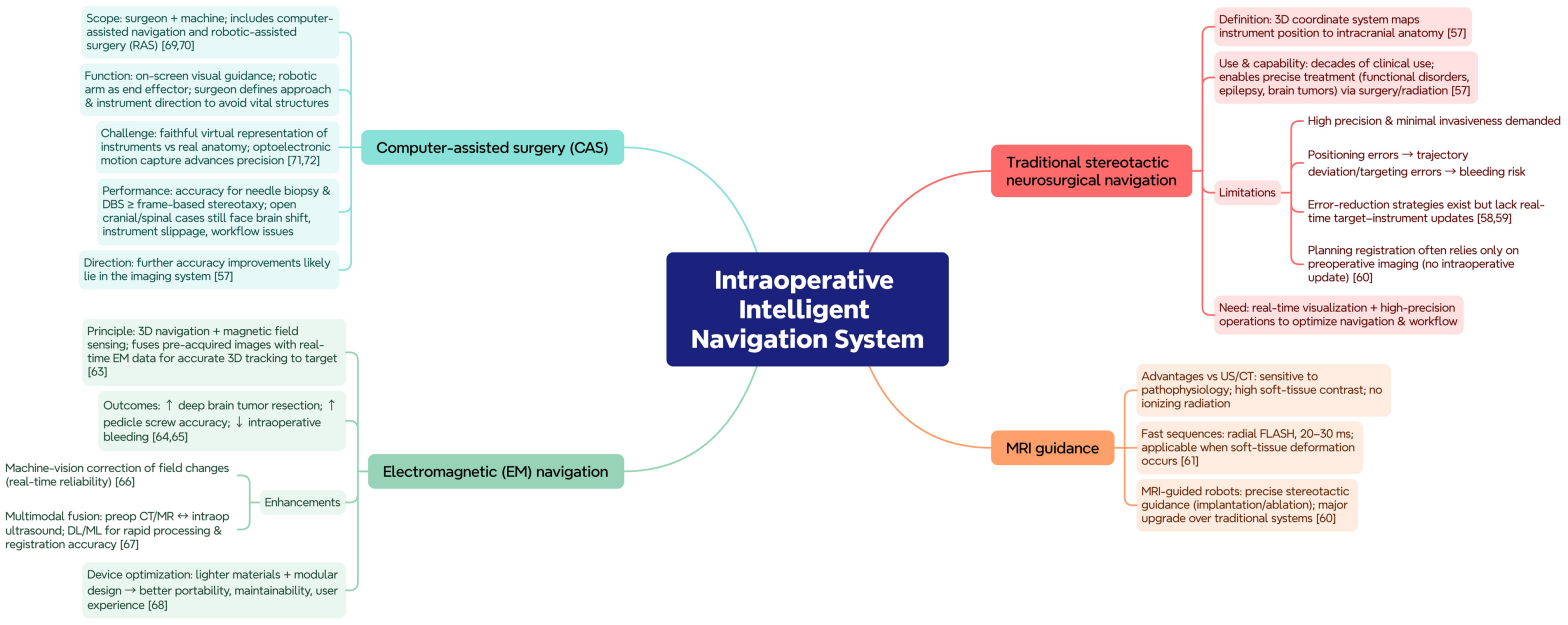
Application framework of intraoperative navigation. Mind-map contrasting traditional stereotactic navigation (limitations without real-time updates) with MRI guidance, electromagnetic navigation, and CAS/RAS. Notes MRI advantages and fast sequences, electromagnetic-based real-time tracking with multimodal/deep learning-enabled enhancements and device optimization, and CAS accuracy with remaining challenges (brain shift, workflow).

Computer-assisted surgery (CAS) is widely defined as a surgical method that involves both the surgeon and the machine, encompassing computer-assisted navigation and robotic-assisted surgery (RAS) (70, 71). Computer-assisted navigation provides visual guidance for surgical instruments displayed on the computer screen. Additionally, robotic-assisted surgery involves a robotic arm that acts as the end effector. The CAS platform enables the surgeon to determine the surgical approach and the direction of instrument manipulation, directing them to the desired anatomical location while avoiding damage to adjacent vital structures. A challenge for navigation systems is to accurately present surgical instruments in virtual space, ensuring they closely match the patient’s real anatomical structures. Advances in optoelectronic motion capture systems have driven the development of high-precision navigation systems (72, 73). While the accuracy of CAS in needle biopsy and deep brain stimulation (DBS) is at least comparable to that of traditional frame-based stereotactic navigation systems, its application in open cranial and spinal surgeries still encounters significant errors associated with brain shift, instrument slippage, and other workflow issues. The optimal direction for improving the inherent accuracy of CAS may lie in the imaging system (58) (Figure 6).

### 3.5 Robot-assisted surgery

Robotics is a fast-growing applied field that is now closely integrated with advancements in machine learning and artificial intelligence, transforming clinical applications in neurosurgery. Doctors and researchers hope that the application of robotics will eliminate mechanical errors during surgery, shorten operation time, and achieve the same or even higher efficiency in minimally invasive surgeries. Although some positive progress has been made in this area, there is still a long way to go due to the complexity of brain anatomy and the inherent spatial limitations of neurosurgical procedures (74). Although many robotic systems are applied in neurosurgery, they can generally be classified into three categories: (1) the telesurgical robot (master–slave). In this type of robot, the surgeon remotely controls the robot’s movements. In this type of robot, the NeuroArm (University of Calgary) is a promising example (75). It is an MRI-compatible robotic arm that can mimic the surgeon’s hand (76). This is the first robot capable of providing haptic feedback and controlled by neurosurgeons, allowing the surgeon to operate from a remote workstation outside the operating room. Reportedly, this robot has been involved in more than 1,000 neurosurgical surgeries, including MRI-guided tumor biopsies, microsurgical dissection, and hematoma evacuation (77). (2) The supervisory surgeon-controlled robot. These devices are primarily used for performing frame-based or frame-free stereotactic tasks, and their applications have expanded from guiding biopsy needles and deep electrodes to planning and inserting spinal pedicle screws. Supervised robots like SpineAssist (78) and Renaissance system (Mazor Surgical Technologies, Caesarea, Israel) (79) are now widely applied in spinal surgeries and have recently been approved for use in brain surgeries (74). (3) Handheld shared/controlled systems. This type of robot is controlled collaboratively by the surgeon and the robot during the operation. Therefore, the robot’s precise movements can be combined with the neurosurgeon’s operational skills and hand flexibility. In a way, this appears to be a win-win situation (74). Due to various reasons, there are currently very few such systems under development. The Steady Hand System (Johns Hopkins University, Maryland, USA) is a typical example (80). The device is held jointly by the surgeon and the robot, allowing for more precise dissection and eliminating hand tremors and muscle fatigue. The NeuRobot (Shinshu University, Matsumoto, Japan) is a microsurgical manipulation system with a rigid neuroendoscope and three micromanipulators, designed for minimally invasive and remotely controlled neurosurgery. The system can perform complex surgeries through a small window with a diameter of 10 mm (81).

**Figure 7:**
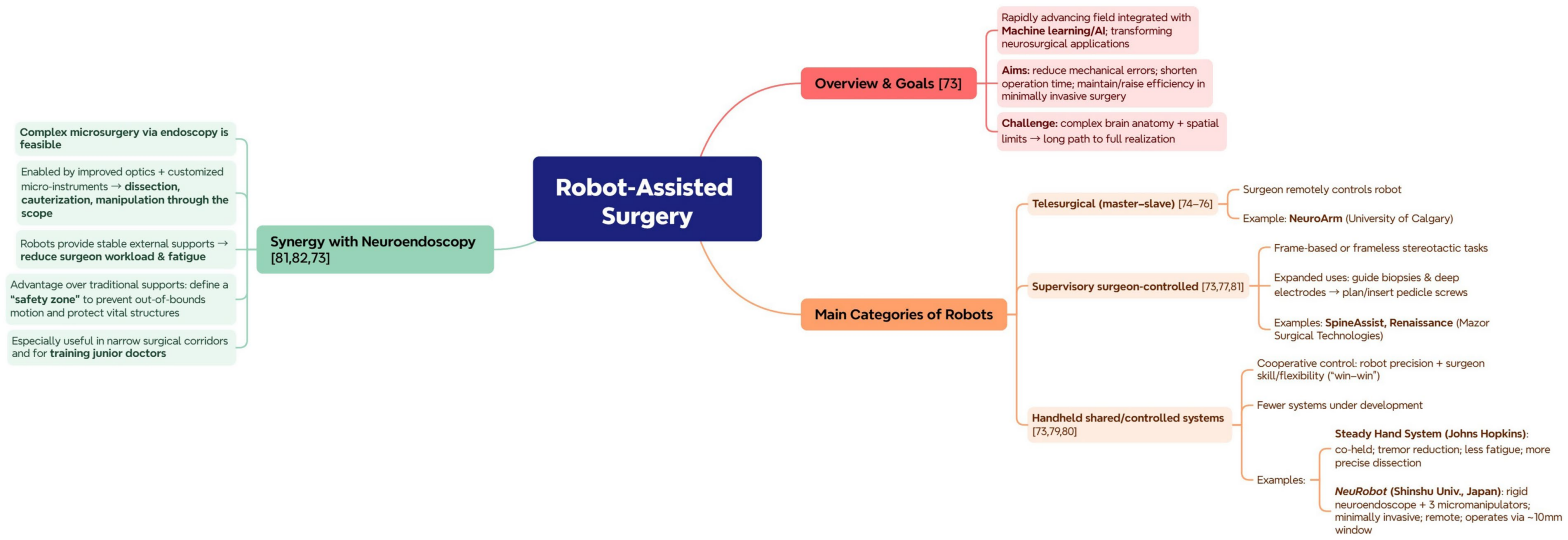
Application framework of robot-assisted surgery. Mind-map highlighting aims (fewer errors and shorter operating time), three robot categories (telesurgical; supervisory surgeon-controlled; handheld shared/controlled) with representative examples, and neuroendoscopic synergy (stable support, “safety zone,” usefulness in narrow corridors and training).

The combination of surgical robots and neuroendoscopy technology is expected to bring significant advancements. Previous studies have shown that performing complex microsurgical neurosurgery through endoscopy is feasible. This progress is due to advancements in optical technology and the development of customized micro-instruments, which have made tissue dissection, cauterization, and manipulation through the endoscope possible (82, 83). At present, robots are primarily used to create stable and reliable external supports, relieving the workload on the surgeon’s hand and reducing muscle fatigue. The advantage of robotic external supports, compared to traditional endoscopic supports, is that they can define a “safety zone,” preventing the surgeon from accidentally damaging vital structures by operating beyond the intended area. This is particularly useful in neurosurgery, as almost all procedures are performed through narrow passages. This function is particularly useful when training junior doctors (74) (Figure 7).

### 3.6 AI

The concept of “artificial intelligence” is currently widely applied in various fields of science and medicine. Technically, AI is a mathematical technique that can automatically solve intellectual problems traditionally addressed by humans (84). The potential of these technologies is in automating medical processes, especially in disease diagnosis, clinical decision-making, and treatment outcome prediction (85). It is expected that the “computer at the bedside” will accelerate clinical decision-making, improve decision reliability, and enhance the quality and safety of healthcare services (86).

The most promising advantage of AI technology is its ability to utilize all available information (even if it exists in unstructured forms, such as images or text) and uncover complex and important patterns (86). Neurosurgery primarily focuses on the surgical treatment of neurological diseases (such as those affecting the brain, spinal cord, and peripheral nervous system), and during this process, a wide range of electronic components, such as intraoperative navigation devices and electronic monitoring equipment, are commonly used to collect and store patient medical data. The routine use of these devices and more advanced digital medical information systems generates a vast amount of real, collectable raw data in neurosurgical clinical studies. These factors make neurosurgery an ideal field for the successful application of AI technology.

Currently, the evidence base for the performance, safety, and economic feasibility of AI methods in neurosurgery is gradually being established. First, in neuro-navigation, a critical challenge of intraoperative navigation is high-fidelity image registration, where preoperative imaging data is updated to reflect real-time changes in the surgical area while planning the navigation. Currently, efforts are underway to develop new machine learning (ML) methods to enhance traditional navigation systems (87). This improvement in image registration technology enhances the speed and accuracy of neurosurgical procedures (88). Second, the topic of Subtask Automation is addressed. Although neurosurgery requires highly skilled surgeons, some tasks can be automated, allowing the doctor to focus on critical areas. Padoy and Hager (89) have shown this doctor–robot interaction model through Hidden Markov models. Similarly, Hu et al. (90) noted that the RAVEN II surgical robot can perform brain tumor ablation semi-autonomously through manual modeling and the implementation of a behavior tree framework. Furthermore, surgical path planning using ML methods in stereotactic brain biopsy procedures can also reduce the risk of damaging blood vessels (91). Finally, ML has shown progress in areas such as intraoperative identification of brain functional regions (92), improvement of motor symptoms in Parkinson’s patients (93), differentiation of intracranial tumors (94), and identification of tumor boundaries (95).

In a systematic review conducted by Senders et al. (96), the authors described 23 studies that evaluated the utility of AI technology in addressing diagnostic and prognostic tasks and compared it to the accuracy of medical judgment in neurosurgery. The results indicated that when machine learning (ML) is used as a complement to expert work, this combination is more effective than relying on either machine learning or clinical experts alone. This result further confirms the hypothesis about the potential applications of AI in neurosurgery (86).

**Figure 8:**
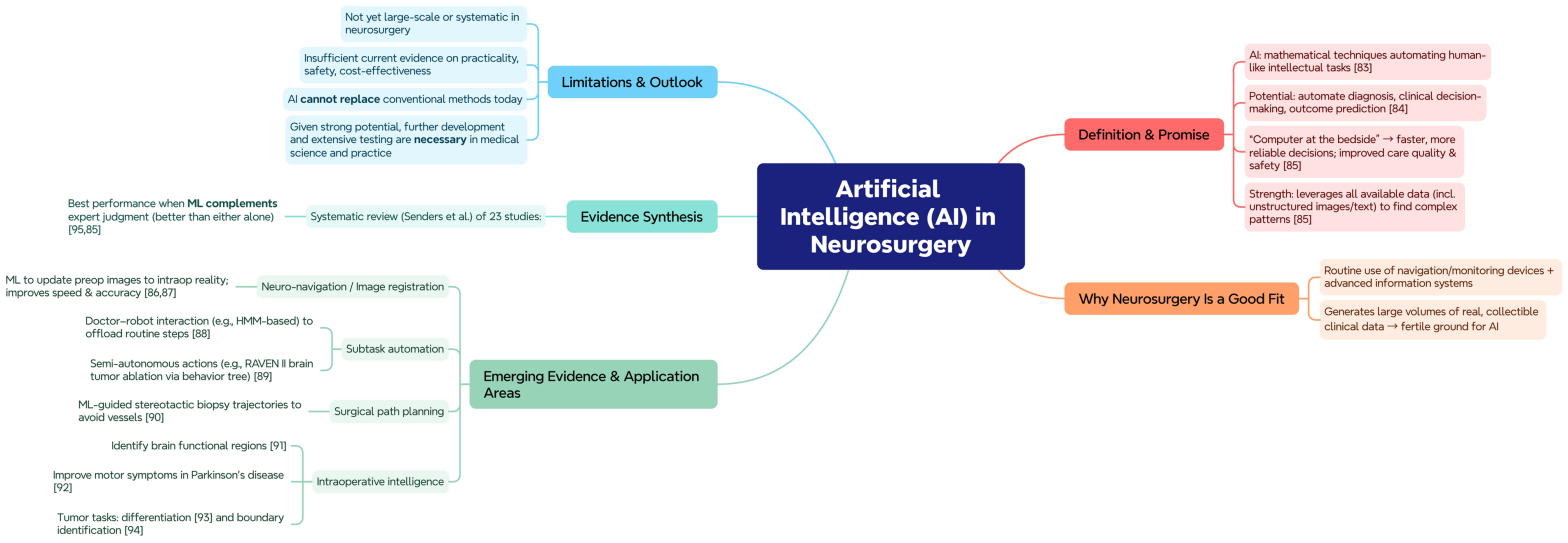
Application framework of AI. Mind-map outlining definition/promise, why neurosurgery is data-rich for AI, key evidence (machine learning + experts outperform either alone), emerging applications (registration, subtask automation, path planning, intraoperative intelligence), and present limitations/outlook.

Despite the rapid development of AI technology, ML, and related big data analysis methods in recent years, these technologies have not yet seen large-scale, systematic application in the field of neurosurgery. Therefore, there is not enough evidence to determine the practicality, safety, and economic feasibility of AI technology in the field of neurosurgery. We cannot assume that AI technology can fully replace conventional medical methods today. However, given the strong potential of AI, we can certainly advocate for the necessity of developing and extensively testing these methods in medical science and practice (Figure 8).

## 4 Discussion

Across the results, the six technologies operate as a connected perioperative system rather than isolated tools. Digital 3D reconstruction converts multimodal images into patient-specific 3D models—the informational “cornerstone” that clinicians use for diagnosis and surgical planning, with clear advantages in spatial understanding and real-time interpretability. These models are then materialized by 3D printing into presurgical anatomic replicas and intraoperative guides that translate virtual plans into precise, task-specific instruments at the bedside. Intraoperative intelligent navigation aligns the preoperative model with the evolving operative field, and—when paired with fast intraoperative MRI and electromagnetic navigation—counteracts brain shift and improves tracking accuracy and safety. Execution is further supported by robot-assisted surgery, including MRI-guided platforms that deliver stereotactic precision for implantation and ablation in complex procedures. Artificial intelligence threads through these stages by enhancing registration, sub-task automation, and decision support, thereby reducing error propagation across the workflow. Tying the components together, the DT/DTT provides a longitudinal, data-centric scaffold that fuses multimodal inputs for preoperative planning, intraoperative adaptation, and postoperative evaluation—enabling the system to evolve from static assistance to adaptive collaboration as data accumulate.

This review was designed as a scoping review to map, rather than adjudicate, the effectiveness of digital technologies across neurosurgical care. Despite a comprehensive search and dual-reviewer screening, marked heterogeneity across interventions, comparators, and reporting scales precluded meaningful pooling. Many reports were single-arm technical evaluations or used surrogate engineering metrics not directly translatable to clinical endpoints. The auxiliary technologies discussed in this review are currently the key cornerstones of digital neurosurgery. The collaborative integration of these technologies, or the dominance of individual technologies in specific contexts, has collectively accelerated the rapid advancement of modern neurosurgery. Given diverse interventions, endpoints, and contexts (clinical vs. educational), quantitative pooling would risk conceptual incoherence and ecological bias. A structured narrative approach better preserves use-case granularity while still enabling cross-domain synthesis.

With the continuous maturation of key technologies in digital neurosurgery and their deeper clinical applications, its future development is expected to present trends of multi-technology integration, intelligent upgrades, and expansion of clinical applications. The following aspects deserve particular attention:

First, multimodal data fusion and intelligent analysis will become the core direction of digital neurosurgery development. At present, different types of imaging data still face difficulties in fusion and limited registration accuracy. In the future, with the help of more advanced AI algorithms (such as Transformers and Federated learning), seamless integration and dynamic modeling of multi-source heterogeneous medical data are expected, thereby constructing a more comprehensive and accurate “patient DT” to provide more reliable evidence for preoperative planning, intraoperative navigation, and postoperative evaluation.

Second, real-time dynamic feedback and adaptive surgical systems are the key for digital neurosurgery to transition from “static assistance” to “dynamic collaboration.” Current intraoperative navigation systems are still limited by dynamic factors such as brain shift and instrument errors. In the future, by combining high-resolution intraoperative imaging with real-time data processing, surgical systems will achieve dynamic updates and automatic corrections of intraoperative models, further improving surgical precision and safety. At the same time, with the integration of extended reality technologies, surgeons will be able to more intuitively perceive the relationship between the surgical area and surrounding critical structures, achieving true “transparent surgery.”

Third, deeper integration of robotics and automation will advance neurosurgery toward higher precision and minimally invasive techniques. Today’s robotic systems largely remain “assistive,” but future robots, with improved closed-loop control (perception-decision-execution), will achieve greater autonomy, such as performing delicate procedures like vascular anastomosis or tissue removal in specific contexts. Moreover, modular, lightweight, and affordable robotic systems will gradually proliferate, allowing more institutions to perform high-precision neurosurgical operations.

Fourth, the role of artificial intelligence in prognosis prediction and personalized treatment will become more prominent. Machine learning models based on big data can be used not only for intraoperative assistance but also for preoperative risk stratification, postoperative complication prediction, and optimization of rehabilitation plans. For instance, by analyzing multi-center, long-term clinical data, AI models can generate personalized treatment pathways and follow-up strategies for each patient, truly achieving end-to-end digital empowerment “from treatment to health management.”

Finally, the establishment of standardized, ethical, and regulatory systems is the safeguard for the sustainable development of digital neurosurgery. At present, new technologies such as DT and AI-assisted diagnosis still lack unified evaluation standards and clinical guidelines. In the future, national drug regulatory agencies (such as the NMPA and FDA) need to accelerate the development of approval processes and technical specifications for digital medical technologies, while also establishing cross-agency and cross-regional data sharing and privacy protection mechanisms to ensure that technological innovation advances within a compliant, safe, and transparent framework.

In conclusion, digital neurosurgery is at a critical stage of transition from technological exploration to large-scale application. In the future, through deep integration of medicine and engineering, driven by clinical needs and empowered by iterative technologies, digital neurosurgery is expected to truly achieve the leap from an “assistive tool” to an “intelligent partner,” ultimately promoting neurosurgical diagnosis and treatment toward greater precision, intelligence, and humanization.

## 5 Conclusion

Digital neurosurgery should be viewed as a unified, data-centric system rather than a set of discrete tools. Across the evidence synthesized, 3D reconstruction/printing turns imaging into patient-specific models and guides; image-guided navigation with intraoperative imaging counters brain shift at the point of action; robotics executes with steadiness in constrained corridors; and AI supports registration, prediction, and workflow orchestration. Their value compounds when information flows seamlessly among them, with the emerging patient “DT” providing a longitudinal scaffold for planning, real-time adaptation, and postoperative learning. Collectively, studies report gains in precision, efficiency, training realism, and communication, although heterogeneity and scarce head-to-head trials temper certainty. To convert promise into generalizable benefit, priorities include interoperable data standards, prospective multi-center evaluation with transparent reporting, and proportionate governance—advancing the field from assistive tools toward an intelligent, adaptive partner in neurosurgical care. As a scoping review, these findings delineate where rigorous systematic reviews and prospective comparative studies are most needed to translate promise into generalizable benefit.

## Footnotes

1
https://pubmed.ncbi.nlm.nih.gov/

2
https://webofscience.com

3
https://www.cnki.net/
